# Screening for an Underlying Tubulopathy in Children With Growth Failure, Simply Maths?

**DOI:** 10.3389/fped.2022.902252

**Published:** 2022-07-14

**Authors:** Caroline Becue, Britt Ceuleers, Marieke den Brinker, Ines Somers, Kristien J. Ledeganck, Hilde Dotremont, Dominique Trouet

**Affiliations:** ^1^Faculty of Medicine and Health Sciences, University of Antwerp, Antwerp, Belgium; ^2^Laboratory of Experimental Medicine and Pediatrics and Member of the Infla-Med Centre of Excellence, University of Antwerp, Antwerp, Belgium; ^3^Department of Pediatrics, Antwerp University Hospital, Edegem, Belgium

**Keywords:** short stature, children, tubulopathy, genetic screening, urinary fractional excretion of electrolytes

## Abstract

**Background:**

Involving pediatric nephrological input in the clinical diagnostic work-up of children with short stature, gave rise to the hypothesis that the presence of an underlying renal tubular disorder in children with short stature is possibly underestimated. This study focussed on the added value of calculated urinary fractional excretion (FE) in the early detection of tubular disorders in children with growth failure.

**Methods:**

This trial was designed as an observational study analyzing the medical files of children between 5 and 16 years who had been referred for short stature to the pediatric endocrinology outpatient clinic at the University Hospital Antwerp between 25/01/2015 and 01/03/2019. Based on the laboratory results of the simultaneously taken blood and urine sample, the fractional excretions of Sodium, Chloride, Potassium, Calcium, Phosphate, and Magnesium were calculated.

**Results:**

Of the 299 patients, 54 patients had at least one deviating fractional excretion value, requiring further investigation (control sample of blood and urine, kidney ultrasound or 24 h urine collection). Genetic screening for tubulopathies was performed in 19 patients. In 5 patients (1.7% of the total population) a tubulopathy was confirmed based on genetic analysis.

**Conclusion:**

This study explored the possibility of using fractional excretions as a screening test to obtain an earlier diagnosis of tubular disorders in children with short stature. Of the 299 patients, 5 patients were diagnosed with a genetically confirmed tubulopathy. Based on these results, we propose a flowchart for an additional work-up in all children with a deviating fractional excretion.

## Introduction

Diagnostic evaluation of growth is necessary for every child with a height less than 2 standard deviation score (SDS) below the mean for age, a decline in height curve crossing two centile lines or a significant discrepancy between the child’s centile and mid-parental centile. The majority of causes underlying growth failure are of non-endocrine origin (such as constitutional delay in growth, familial short stature and systemic disease). In only 5% of the cases, short stature has a pure endocrine cause. If children grow slowly without an obvious explanation through history or clinical examination, conditions such as renal tubular disorders should be considered. The current literature confirms that any defect along the tubule can potentially hamper growth, and that a specific treatment of the defective tubular reabsorption can result in significant catch-up growth (1–3). In most tubular disorders, the combination of electrolyte supplementation, dietary adjustments and eventual therapeutic inhibition of excessive tubular excretions (by indomethacin and/or ACE-inhibition) results in the best outcome of growth.

Renal tubular disorders are a rare and heterogeneous group of diseases. The non-specific clinical and biochemical presentation contributes to the frequent delay in diagnosing children with tubular dysfunctions. Nevertheless, all of them have certain electrolyte and volume disturbances due to the dysfunction of tubular reabsorption. The clinical presentation depends on the specific part of the tubular function that is affected, but a tubular defect can be overlooked for years. The first symptoms in children with renal tubular disorders are often non-specific such as fatigue, mild growth retardation, impaired physical activity, vomiting, constipation and mild polyuria or polydipsia (4–10). Mere blood biochemistry is often insufficient to diagnose young children with an underlying tubular disorder, since mild disturbances in serum electrolytes may often be disregarded at young ages. Therefore, a biochemical urinalysis should be paired with blood biochemistry in order to detect abnormally high fractional excretions (11).

Current guidelines on the standard diagnostic approach in children with growth failure do not take urinary electrolyte measurements nor calculation of urinary fractional excretions into account. Only two reports in the literature mention basic urinary screening in children with short stature (7, 8). However, this screening comprises only the detection of leucocyturia, hematuria, and proteinuria and therefore it can only exclude underlying urinary tract infections or glomerular disorders.

The only tubular disorder explicitly mentioned in the current guidelines on growth failure is renal tubular acidosis (8). However, by evaluating a blood acid-base equilibrium and no combined urinary biochemistry, only overt renal tubular acidosis can be diagnosed, while incomplete renal tubular acidosis will be overlooked. Moreover, also other tubular dysfunctions such as renal Fanconi syndrome, hypophosphatemic rickets, Bartter syndrome, and Gitelman’s disease, as well as nephrogenic diabetes insipidus might impede growth (6, 9).

## Aim

In this study we hypothesized that renal tubular disorders in children with short stature are possibly underestimated. Currently, systematically performed urinary electrolyte measurement in children with growth failure is not yet part of the standard diagnostic endocrinological work-up.

Therefore, in this study we aimed to evaluate whether the implementation of calculating the fractional excretion of electrolytes in the classical diagnostic screening protocol of growth failure improves the yield of finding an underlying diagnosis and whether this may lead to a more tailored treatment of growth failure.

## Materials and Methods

### Population

This research was designed as an observational study analyzing the medical files of children between 5 and 16 years who had been referred for short stature to the pediatric endocrinology outpatient clinic at the University Hospital Antwerp for between 25/01/2015 and 01/03/2019. Short stature was defined as a height of less than 2 standard deviations under the mean height for age, gender, and population (8). Children previously diagnosed with a known chronic disease were excluded. In total, the medical files of 299 patients were analyzed in this study. All data were de-identified only registering their date of birth.

### Study Design

All 299 patients had been screened during their first visit following the standard workup. According to this standard procedure, in case of abnormal blood results, a further endocrinological diagnostic protocol was performed, and in case of dysmorphic growth (defined as a deviated proportion in arm length span, sitting height, standing height, head circumference, weight, BMI of facial dysmorphic features), karyotyping, and microarray were added to this diagnostic workup.

Parallel to this standard endocrinological and genetic workflow, we evaluated renal tubular function, by calculating the urinary fractional excretions on the standard urine sample and blood test that were routinely collected during this first visit Fractional excretions of electrolytes defined underneath were calculated by the use of the following formula:

The formula used to calculate fractional excretions is:


FE=electrolyt100×urinary⁢concentrate⁢electrolyte ×⁢creatinine plasma⁢concentration⁢electrolyte ×⁢creatinine⁢urine 

The serum Magnesium concentration was multiplied by 0.8 since in children only 80% of the serum magnesium is freely filtered by the glomerulus, with the remaining part being protein-bound.

The major advantage of this method is its simplicity. The calculation of fractional excretions represents the mainstay in screening children suspicious of an underlying tubular disease (5). Normal values of the fractional excretions are based on the handbook of the European Society of Pediatric Nephrology (ESPN) (10). In summary, following values were considered as normal threshold: sodium, chloride, and calcium < 1%; magnesium < 4%; phosphate < 15%, and potassium < 20%.

In the event of aberrant values, the collection and analysis of blood and urine samples was repeated. In case of recurrent abnormal values, referral for an extensive nephrological investigation was initiated, including a focused history questioning, additional metabolic urine analysis (e.g., amino-aciduria, beta-2 microglobulinuria), 24 h urinary collection and ultrasound imaging of bladder and kidneys. If suspicion of an underlying tubular disorder persisted, genetic analysis was performed. Treatment with oral specific supplementation of electrolytes was started in patients suspicious for an underlying tubulopathy.

### Laboratory Measurements

Both in blood and in urine, concentrations of creatinine, potassium, chloride, bicarbonate, calcium, phosphate, and magnesium had been analyzed at the laboratory of biochemistry of the Antwerp University Hospital, Belgium.

Genetic testing was performed by the Institute de Pathologie et de Génétique (IPG) at Gosselies, Belgium. The analysis consisted of a panel containing 37 genes implicated in tubulopathies (Multiplex PCR and Illumina MSeq sequencing). This panel has been developed by the working group for tubulopathies in the European Consortium for High-Throughput Research in Rare Kidney Diseases (EURenOmics) (12).

Tubulopathies can be caused by multiple genes or can have a phenotypic overlap (13), which are covered with this panel. Panel sequencing of selected genes has the advantage of achieving high gene coverage and this at lower costs compared to next-generation sequencing (NGS).

### Definitions

Short stature was defined as a height of less than 2 standard deviations under the mean height for a certain age, gender, and population group (14).

The effectiveness of the treatment was termed as catch-up growth. We used the definition of catch-up growth being a height velocity above the normal limit for age during at least 1 year (15).

### Statistical Analysis

A sample size calculation was performed using a total population of children in Belgium of 50.000 with an expected scatter of 50 and 5% margin of error (16). This resulted in a recommended sample size of 382 patients. Scattered dot plots of the various fractional excretions (sodium, potassium, calcium, phosphor, magnesium and chloride) in relation to the height z-score were created. Hitherto, the height z-scores were first calculated for each participant based on height, sex, and age (17). Next, scattered dot plots were created in SPSS for statistics version 26.0.

## Results

**FIGURE 1:**
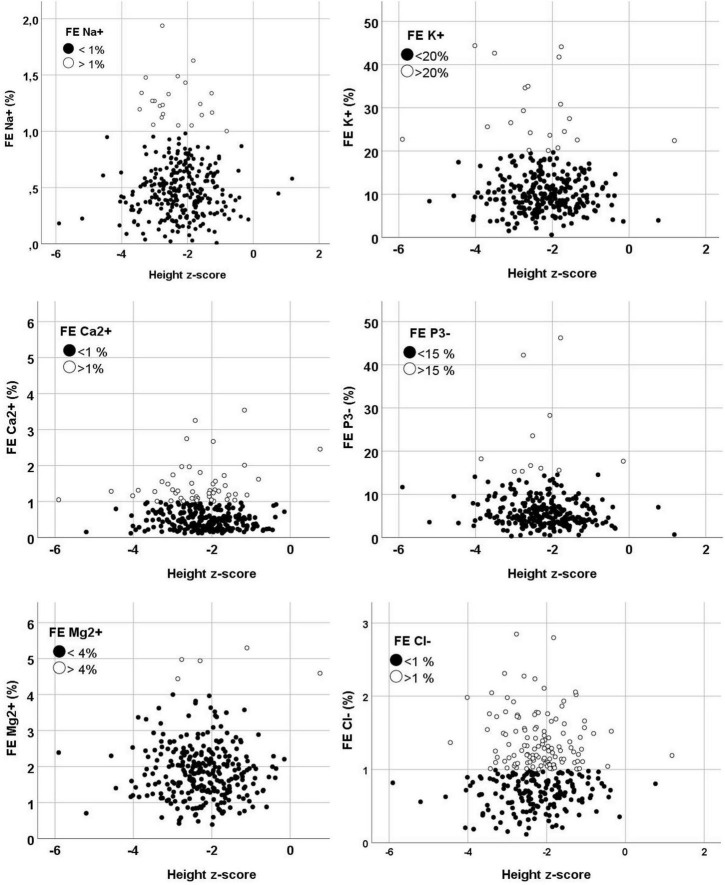
Grouped scatters of the fractional excretion by height z-scores. Normal fractional excretion values are displayed in black dots and abnormal fractional excretion values are displayed in white dots. FE, fractional excretion.

In total 299 patients were included. The mean age at the time of the first urine sample was 10.6 ± 4.1 years. Figure 1 displays the scattered dot plots of the various fractional excretions (sodium, potassium, calcium, phosphor, magnesium and chloride) in relation to the height z-score.

**FIGURE 2:**
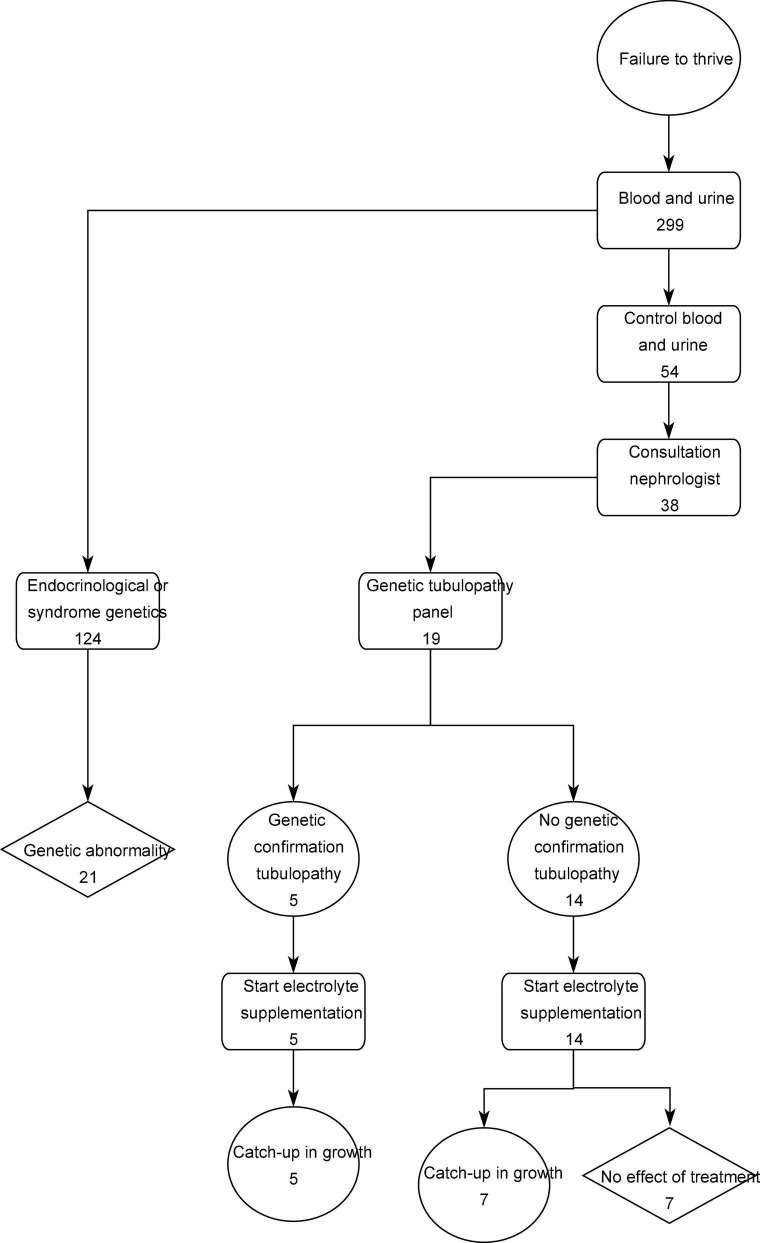
Flow chart.

As shown in the flow chart (Figure 2), in 124 patients of the 299 patients (41.4%), specific genetic testing was performed as part of the standard endocrinological workup, with karyotyping, growth panel sequencing and SHOX analysis.

In 54 of the 299 patients (18.1%) at least one deviant fractional excretion was calculated and control blood and urine samples were taken. Thirty-eight patients (12.8%) of the study population were referred to the pediatric nephrology out-patient clinic and had a renal ultrasound, while 21 of these patients additionally were asked for a 24-h urine collection. Due to one or more persistent aberrant results in this nephrological work-up, genetic testing for tubulopathy was performed in 19 patients (6.4%).

In 5 patients a tubulopathy was diagnosed based on genetic analysis. This corresponds to 1.7% of the total included population and 26.3% of the patients in which panel testing was performed.

**FIGURE 3:**
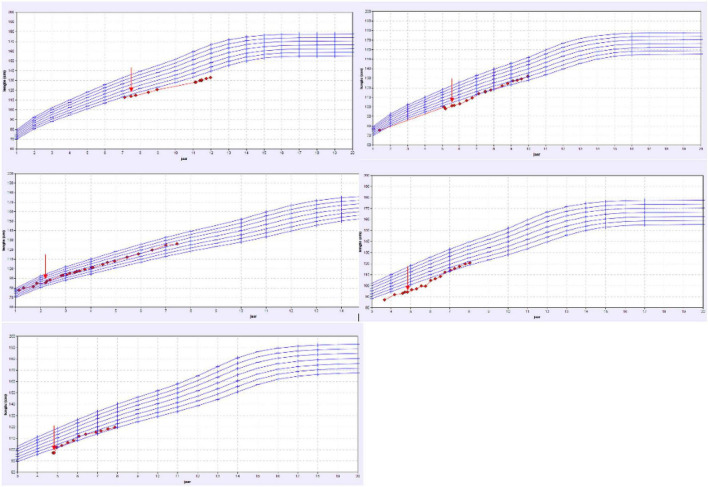
Overview of the growth charts of patients with a proven tubulopathy. X-axis: the age in years and Y-axis: height in cm. The red arrow shows the time point the treatment with specific oral electrolyte supplementation was initiated.

Mutations detected by the tubulopathy gene panel include: a heterozygotic variant c.1639 C > T of the TRPM6 gene, two hypophosphatemic rickets (deletion of Xpter and duplication of Wpter which includes both the SHOX and PHEX genes), one Fanconi syndrome (heterogenetic mutation of the EHHADH gene) and one Gitelman syndrome (heterozygotic mutations in SLC12A3 gene). These five patients had a clear correlation between the genetic mutation and their phenotype with overt electrolyte loss according to the respective calculated fractional excretion. All of them were treated with electrolyte supplements specifically compensating the tubular loss. The 2 patients with SHOX mutation additionally received growth hormone therapy. As illustrated by the growth charts (Figure 3), 4 of these 5 patient showed a catch up growth. The patients characteristics are shown in Table 1.

**TABLE 1:** Overview of the patients’ characteristics with a genetically proven tubulopathy.

Tubulopathy, genetic confirmed											
Subject	Age (y)	Height (cm)	Weight (kg)	BMI (kg/m^2^)	Diagnosis	Mutation	Clinical presentation	Parental height (cm)		Bone age	Pubertal status
								Paternal	Maternal		
1	7.1	112.7	17.2	13.5	Tubulair Mg reabsorption defect	TRPM6	FTT	180	167	Confirm age	A1 P1 M1
2	2.4	86.4	11.3	15.1	Gitelman syndrome	SLC12A3	FTT, salt craving, muscle cramps, decreased muscle strength	196	164	Confirm age	A1 P1 M1
3	5.1	98.2	16.1	16.7	X-linked hypophosphatemic rickets and SHOX mutation	Xp22.33p11.4 deletion (including SHOX and PHEX)	FTT, schisis, dilated left ureter, patent foramen ovale, coeliac disease	171	164	Delayed skeletal age 4y2m	A1 P1 M1
4	3.7	87.1	13.5	17.8	X-linked hypophosphatemic rickets	c.1080-72C > T variant in intron 9 of PHEX gene	FTT, unstable gait, frontal bossing, disproportional body (shorted lower limbs)	180	164	Shows metaphyseal chondrodys plasia, no skeletal age calculated	A1 P1 M1
5	5.0	102.0	14.2	13.6	Autosomal dominant Fanconi Syndrome	EHHADH gene	FTT, cholecystic lithiasis	unknown due adoption		Not available	A1 P1 G1

*Pubertal status, Tanner stadia (A,P,G/M); FTT, failure to thrive; BMI, body mass index.*

Of the remaining 14 patients, an underlying incomplete renal tubular acidosis was diagnosed (by ammonium chloride challenge test) in 3 children and a respiratory-chain defect-induced Fanconi syndrome in 1 child. These patients showed a clear catch-up growth during specific supplementation of minerals. Two patients with severe renal magnesium loss and one child with major tubular potassium-and salt loosing also showed an increased growth velocity during the tailored oral supplementation.

**FIGURE 4:**
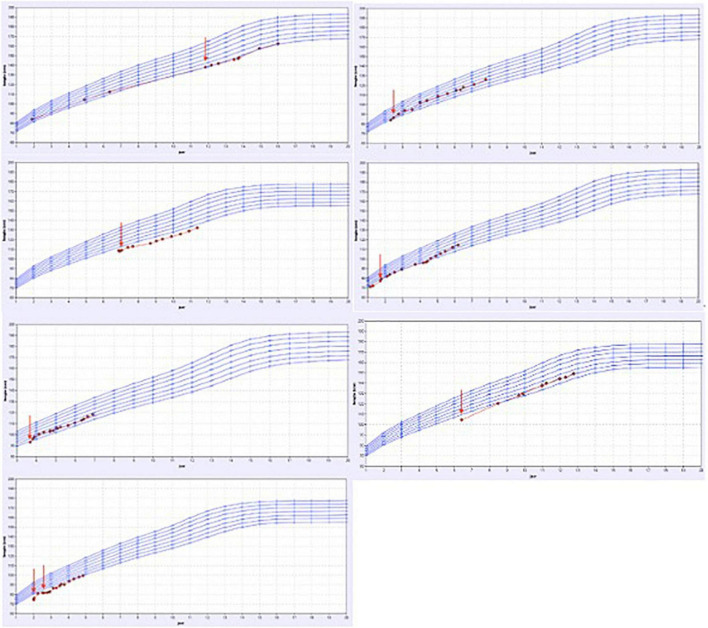
Overview of the growth charts of patients without a proven tubulopathy but who did have a catch-up in growth after treatment. X-axis: the age in years and Y-axis: height in cm. The red arrow shows the time point the treatment with specific oral electrolyte supplementation was initiated.

The growth charts of these patients are presented in Figure 4 and in Table 2, the patient characteristics are represented.

**TABLE 2:** Overview of the patients’ characteristics without a genetically proven tubulopathy.

Tubulopathy, not genetic confirmed											
Subject	Age (y)	Height (cm)	Weight (kg)	BMI (kg/m^2^)	Diagnosis	Supplementation	Clinical presentation	Parental height		Bone age	Pubertal status
								Paternal	Maternal		
1	2.5	86.5	10.92	14.6	Renal tubular acidosis	Iron, vitamine D, sodium bicarbonate	FTT, dysmorphic dental- and hair growth, development delay, periorbital edema, polyuria, ferriprive anemia	/	/	confirm age	A1 P1 G1
2	1.9	79	10.335	16.6	Incomplete renal tubular acidosis	Sodium bicarbonate	FTT, diarrhea, fatigue	172,5	169	delayed skeletal age, 1y3m	A1 P1 G1
3	3.8	93	11.4	13.2	Incomplete renal tubular acidosis	Potassium citrate, sodium bicarbonate, Vitamine D	FTT	/	/	/	A1 P1 G1
4	2.0	74.5	7.85	14.1	Fanconi syndrome secundairy to mitochondrial respiratory chain defect	Potassium and salt supplements, indometacin, nasogastric feeding	FTT, facial dysmorphics, motoric development delay, polyuria, recurrent urine infections by an ectopic kidney	/	/	/	A1 P1 M1
5	11.6	136.7	31.15	16.5	Salt losing nefropathy, hyperkaliuria, hypercalciuria	Salt suppletion (sodium chloride)	FTT, polyuria	unknown due adoption		delayed skeletal age, 10y	A1 P1 G1
6	6.11	108.5	17.7	15	Salt- and magnesium losing nefropathy	Magnesium and sodium chloride	FTT, salt craving	170	153,8	delayed skeletal age, 5y9m	A1 P1 M1
7	9.1	124.4	33.5	22.1	Hypermagnesuria	Magnesium	FTT, born small for gestational age, urine tract infections, reduced muscle strength	167	157	delayed skeletal age, 8y10m	

*Pubertal status, Tanner stadia (A,P,G/M); FTT, failure to thrive; BMI, body mass index.*

In total, irrespective of the genetic results, in 19 patients, compensation of the specific tubular electrolyte losses by tailored oral mineral supplements (such as sodium chloride, sodium bicarbonate, potassium citrate, magnesium oxide, sodium phosphate, or calcium carbonate) had been initiated by the pediatric nephrologist, and in 7 patients, the oral supplementation was discontinued after 12 months due to lack of catch-up growth.

## Discussion

Any tubular disorder in a child can result in impaired growth. These children often present with a borderline or normal blood biochemistry and therefore an underlying tubular disorder can easily be overlooked. Abnormally high fractional excretions can be the missing link to reveal an underlying tubular disorder as the specific cause of short stature in children. In this study, extending the classical screening for children with short stature by calculating urinary fractional excretions, in order to improve the efficiency of detecting an underlying diagnosis, was investigated.

Only two reports in the current literature mention merely basic urinary screening in children with short stature, but do not mention the calculation of urinary fractional excretions (7, 16). Case reports concerning the link between short stature and tubular disorders are scarce, and not a single large-scale study is available on screening of fractional excretions in children with short stature.

The present study was able to compile an extensive number of children with short stature and allowed to calculate the fractional excretions of several electrolytes in a tertiary setting where multidisciplinary follow-up by pediatric endocrinologists and pediatric nephrologists is guaranteed. Tubular dysfunction is very relevant in the differential diagnosis of short stature, however, rather rare, which was confirmed in this study with 3% of the patients to be diagnosed with proven tubular dysfunction. To the best of our knowledge, no other reports are available on incidence of tubular dysfunction in children with growth failure.

**FIGURE 5:**
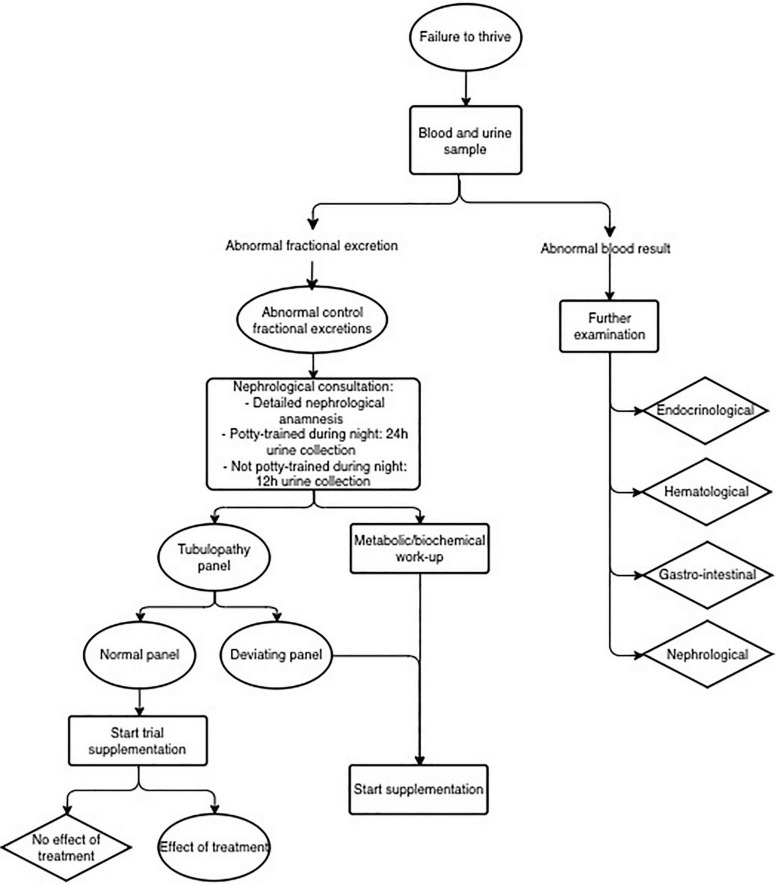
Flow chart for failure to thrive.

Since the fractional excretion is a proxy of tubular dysfunction, one could speculate that the higher the sum of deviating fractional excretions, the higher the chance of an underlying tubular pathology. Confirming this hypothesis would help clinicians making the decision to perform a tubular panel genetic test. Unfortunately, we were not able to confirm this hypothesis in this limited number of patients with one or more abnormal fractional excretion. The genetic tubulopathy panel currently consists of 37 genes, thus not implementing all known causative genes leading to a tubulopathy. Expanding the number of included genes would further increase the diagnostic yield since the majority of tubulopathies has a clear genetic origin. However, the cost efficiency must be kept in mind when considering to expand the panel (18). The results of our current study confirm that the diagnosis of an underlying tubulopathy is not merely genetic, but also depends on the combination of clinical features and laboratory values. A complete work flow focusing on both entities thus remains indispensable, and is presented in Figure 5.

The ultimate confirmation that the diagnosed tubulopathy is directly linked to the short stature, consists in the evidence of catch-up growth during the period of supplementing the patient with the specific minerals linked to the respective tubulopathy. This means that even in the case of a negative genetic screening for tubulopathies, the treating physician might be inclined to treat the patient because of major biochemical abnormalities in the urinary excretion or because of associated clinical symptoms of a tubular disorder. When this treatment gives rise to obvious catch-up growth, the tubulopathy is very likely linked to the short stature. Therefore we also registered these patients in our database. The most probable explanation for this phenomenon is the fact that genetic analysis for tubular disorders is still a relatively new study subject, and therefore not all mutations causing tubular disorders are known at this point or can be genetically confirmed with the same sensitivity. Moreover, the specificity of the genetic screening is 64% (11) and for example a complete deletion of a gene can be missed in this particular screening test. If the clinical and or biochemical suspicion is found to be present, a more thorough genetic diagnostic pathway could be considered.

This study has some limitations. First, we were not able to include the anticipated number of 382 patients that was required according to the calculated sample size. However, we managed to study the files of 299 patients, which is already a representative number. Second, we were not able to calculate the sensitivity or specificity from the available data since the data on the number of false negative patients were lacking. When a patient was considered negative based on the initial screening data, no further investigations were conducted. This way we could not calculate the number of false negative patients. Based on available data we were able to calculate the specificity [true negative/(true negative + false positive)] and the positive predictive value (true positive/true positive + false positive). The specificity was 91.8% and the positive predictive value was 22.2%. From these data we can carefully conclude that the suggested flow chart is able to identify the patients without tubulopathy, however, a larger study including follow up samples from all patients to calculate the sensitivity and negative predictive value is needed.

We propose to continue the current study with expanding the number of patients, and by implying a lower threshold for genetic testing of tubulopathies. Supported by our study results, we propose a new work flow (Figure 5) in which in every patient with at least one deviating fractional excretion, the tubulopathy panel is performed. Although this frequent genetic examination implies a financial investment, we expect it to be cost-effective, considering the huge gain associated with the relatively low-cost treatment. In comparison with the low threshold for endocrinological genetic screening, where almost half of the patients undergoes genetic screening, the tubulopathy gene panel represents only a small fraction. With a well-considered genetic screening request (as proposed in the flowchart displayed in Figure 5), this might even have a relatively higher yield. In the present study, 16.9% of the patients had a deviating genetic result.

When considering the implementation of calculating fractional excretions into the protocol of children presenting with short stature, the number of children being tested for tubular disorders might increase, thereby risking more second biochemical testing in case of false positive results. However, the treatment of tubular disorders such as electrolyte administration is a minor intervention compared to other more invasive treatments such as growth hormone treatment. (19)

To conclude, we propose a flowchart (Figure 2) as an adapted guideline for pediatricians in the diagnostic approach of children with short stature, based on the diagnostic yield of tubular disorders in children with growth failure in this current pilot study. A prospective study will be required to confirm our findings in children referred for short stature evaluation.

## Data Availability Statement

The data analyzed in this study is subject to the following licenses/restrictions: Confidentiality of patient files. Requests to access these datasets should be directed to corresponding author.

## Ethics Statement

The studies involving human participants were reviewed and approved by the Ethics Committee Antwerp University Hospital. Written informed consent to participate in this study was provided by the participants or their legal guardian/next of kin.

## Author Contributions

All authors listed have made a substantial, direct, and intellectual contribution to the work, and approved it for publication.

## Conflict of Interest

The authors declare that the research was conducted in the absence of any commercial or financial relationships that could be construed as a potential conflict of interest.

## Publisher’s Note

All claims expressed in this article are solely those of the authors and do not necessarily represent those of their affiliated organizations, or those of the publisher, the editors and the reviewers. Any product that may be evaluated in this article, or claim that may be made by its manufacturer, is not guaranteed or endorsed by the publisher.
