# Supplementary material for: Screening for an Underlying Tubulopathy in Children With Growth Failure, Simply Maths?
Source: Front Pediatr. 2022 Jul 14;10:902252. doi: 10.3389/fped.2022.902252 (PMC9334702; doi:10.3389/fped.2022.902252)
Supplement: Supplementary file 1 [file Data_Sheet_1.PDF]

Table 1 Descriptive statistics

	N	Minimum	Maximum	Mean	Std. Deviation
Age at inclusion	299	0,266	17,265	10,581	4,121
FE Na	299	0,008	1,938	0,527	0,302
FE K	297	0,606	44,375	11,367	6,547
FE Cl	299	0,114	2,849	0,952	0,462
FE Ca	298	0,119	3,540	0,656	0,492
FE P	298	0,343	46,239	6,443	4,911
FE Mg	298	0,389	5,301	1,902	0,841
Follow-up FE Na	54	0,101	2,148	0,555	0,363
Follow-up FE K	54	0,129	39,735	12,075	7,396
Follow-up FE Cl	54	0,264	2,572	0,965	0,448
Follow-up FE Ca	54	0,124	3,493	0,775	0,659
Follow-up FE P	54	0,105	17,590	6,379	3,687
Follow-up FE Mg	54	0,190	4,324	2,019	0,918
