# Supplementary material for: Screening for an Underlying Tubulopathy in Children With Growth Failure, Simply Maths?
Source: Front Pediatr. 2022 Jul 14;10:902252. doi: 10.3389/fped.2022.902252 (PMC9334702; doi:10.3389/fped.2022.902252)
Supplement: Supplementary file 2 [file Data_Sheet_2.PDF]

Tubulopathy, genetic confirmed											
Subject	Age (y)	Height (cm)	Weight (kg)	BMI (kg/m²)	Diagnosis	Mutation	Clinical presentation	Parental height (cm)		Bone age	Pubertal status
								Paternal	Maternal		
1	7.1	112.7	17.2	13.5	Tubulair Mg reabsorptie defect	TRPM6	FTT	180	167	confirm age	A1 P1 M1
2	2.4	86.4	11.3	15.1	Gitelman syndrome	SLC12A3	FTT, salt craving, muscle cramps, decreased muscle strength	196	164	confirm age	A1 P1 M1
3	5.1	98.2	16.1	16.7	X-linked hypophosphatemic ricketts and SHOX mutation	Xp22.33p11.4 deletion (including SHOX and PHEX)	FTT, schisis, dilated left urether, patent foramen ovale, coeliac disease	171	164	delayed skeletalage 4y2m	A1 P1 M1
4	3.7	87.1	13.5	17.8	X-linked hypophosphatemic ricketts	c.1080-72C>T variant in intron 9 of PHEX gene	FTT, unstable gait, frontal bossing, dysproportional body (shorted lower limbs)	180	164	shows metaphyseal chodrodysplasia, no skeletal age calculated	A1 P1 M1
5	5.0	102.0	14.2	13.6	Autosomal dominant Fanconi Syndrome	EHHADH gene	FTT, cholecystic lithiasis	unknown due adoption		Not available	A1 P1 G1

Tubulopathy, not genetic confirmed											
Subject	Age (y)	Height (cm)	Weight (kg)	BMI (kg/m²)	Diagnosis	Supplementation	Clinical presentation	Parental height		Bone age	Pubertal status
								Paternal	Maternal		
1	2.5	86,5	10,92	14,6	renal tubular acidosis	Iron, vitamine D, sodiumbicarbonate	FTT, dysmorfic dental- and hair growth, development delay, periorbital oedema, polyuria, ferriprive anemia	/	/	confirm age	A1 P1 G1
2	1.9	79	10,335	16,6	incomplete renal tubular acidosis	sodium bicarbonate	FTT, diarhea, fatigue	172,5	169	delayed skeletal age, 1y3m	A1 P1 G1
3	3.8	93	11,4	13,2	incomplete renal tubular acidosis	potassium citrate, sodium bicarbonate, Vitamine D	FTT	/	/	/	A1 P1 G1
4	2.0	74,5	7,85	14,1	Fanconi syndrome secundairy to mitochondrial respiratory chain defect	potasium and salt supplements, indometacine, nasogastric feeding	FTT, facial dysmorfics, motoric development delay, polyuria, recurrent urine infections by an ectopic kidney	/	/	/	A1 P1 M1

5	11.6	136,7	31,15	16,5	salt losing nefropathy, hyperkaliuria, hypercalciuria	Salt suppletion (sodium chloride)	FTT, polyuria	unknown due adoption		delayed skeletal age, 10y	A1 P1 G1
6	6.11	108,5	17,7	15	Salt- and magnesium losing nefropathy	Magnesium and sodium chloride	FTT, salt craving	170	153,8	delayed skeletal age, 5y9m	A1 P1 M1
7	9.1	124,4	33,5	22,1	hypermagnesuria	Magnesium	FTT, born small for gestational age, urine tract infections, reduced muscle strength	167	157	delayed skeletal age, 8y10m	
